# Real-World Experience of Ceftobiprole for Community- and Hospital-Acquired Pneumonia from a Stewardship Perspective

**DOI:** 10.3390/microorganisms12040725

**Published:** 2024-04-03

**Authors:** Silvia Corcione, Ilaria De Benedetto, Massimiliano Carlin, Emanuele Emilio Pivetta, Silvia Scabini, Cecilia Grosso, Nour Shbaklo, Massimo Porta, Enrico Lupia, Francesco Giuseppe De Rosa

**Affiliations:** 1 Department of Medical Sciences, Infectious Diseases, University of Turin, 10126 Turin, Italy; ilaria.debendetto@edu.unito.it (I.D.B.); silviascabini88@gmail.com (S.S.); cecilia.grosso@unito.it (C.G.); nour.shbaklo@edu.unito.it (N.S.); francescogiuseppe.derosa@unito.it (F.G.D.R.); 2 Division of Geographic Medicine, Tufts University School of Medicine, Tufts University, Boston, MA 02111, USA; 3 Department of Medical Sciences, Internal Medicine, University of Turin, 10126 Turin, Italy; massimiliano.carlin@gmail.com (M.C.); massimo.porta@unito.it (M.P.); 4 Department of Medical Sciences, Emergency Medicine, University of Turin, 10126 Turin, Italy; emanuele.pivetta@unito.it (E.E.P.); enrico.lupia@unito.it (E.L.)

**Keywords:** ceftobiprole, antimicrobial stewardship, carbapenem-sparing, glycopeptide-sparing, internal medicine

## Abstract

Ceftobiprole is a fifth-generation cephalosporin approved by European and American regulatory agencies for the treatment of community-acquired pneumonia (CAP) and hospital-acquired pneumonia (HAP). Ceftobiprole administration is useful in severe CAP as well as HAP where the potential is to save other β-lactams including carbapenems or linezolid/vancomycin in clinical practice. The aim of this study was to report the real-world evidence of ceftobiprole in patients with CAP and HAP in a single center. In this retrospective study, we included 159 patients with CAP or HAP: 105 (66%) had CAP and 54 (34%) had HAP. The median age was 70 years (IQR 60–77), the median Charlson Comorbidity Index was 5 (IQR 3–7.5) and baseline INCREMENT ESBL score was 8 (IQR 6–11). Ceftobiprole was mostly given as a combination treatment (77%) or as a carbapenem-sparing strategy (44%). There were no differences in mortality between shorter and longer duration of treatment (<7 days compared with ≥7 days (HR 1.02, C.I. 0.58–1.77, *p* = 0.93) or between first-line (HR 1.00, C.I. 0.46–2.17, *p* = 0.989) and second-line therapy. Ceftobiprole use in CAP or HAP in the real world is effective as a first- and second-line treatment as well as a carbapenem-sparing strategy. Further studies are needed to explore the full potential of ceftobiprole, including its real-world use in antimicrobial stewardship programs.

## 1. Introduction

Ceftobiprole is a fifth-generation cephalosporin approved by European Medicines Agency (EMA) and the Food and Drug Administration (FDA) for the treatment of community-acquired pneumonia (CAP) and hospital-acquired pneumonia (HAP) [1,2]. Ceftobiprole has shown non-inferiority versus ceftriaxone in a double-blinded, multicenter, randomized trial with linezolid in case of high risk of methicillin-resistant *Staphylococcus aureus* (MRSA) or ceftriaxone-resistant *Streptococcus pneumoniae* in CAP [1,3] and in a double-blinded, multicenter, randomized study versus ceftazidime with linezolid in HAP [2]. It has a bactericidal activity against Gram-positive bacteria, including MRSA, most Enterobacterales, and some *Pseudomonas aeruginosa* strains, whereas it is usually not active against Gram-negative bacteria producing extended-spectrum β-lactamases (ESBL), serine and metallo-carbapenemases [4,5,6,7,8].

More precisely, the in vitro activity of ceftobiprole demonstrates potent binding against PBPs of Gram-positive bacteria (GPB), including those with decreased β-lactam sensitivity, such as PBP2x and PBP2b in PRSP, and PBPa, which confers methicillin resistance in *S. aureus* strains. In vitro bactericidal activity against MRSA strains showed similar or superior kinetics to those of vancomycin and linezolid. Furthermore, recent findings from phase III ceftobiprole SSTIs and pneumonia clinical trials showed promising activity on MRSA isolates, including Panton–Valentine-leukocidin-positive strains, with a slight variation according to SCC*mec* or clone type [9].

As previously mentioned, ceftobiprole retains activity against a wide spectrum of Gram-negative bacteria (GNB) and is stable against a wide variety of β-lactamases. Being similar to ceftriaxone, cefepime, and ceftazidime [9]; the class A β-lactamases PC1 staphylococcal penicillinase, TEM, some SHV types, and the K1 β-lactamase of *Klebsiella oxytoca* have no lytic enzymatic action against ceftobiprole. The drug is degraded by both extended-spectrum β-lactamases (ESBLs) and serine-carbapenemases. Class B, several class C chromosomal AmpC-type β-lactamases, and some class D β-lactamases, have lytic action on ceftobiprole’s structure [9].

Ceftobiprole use in severe CAP relies on its MRSA activity and Gram-negative coverage, even on monotherapy, such as post-influenza bacterial pneumonia or COVID-19-related cases, especially in elderly and comorbid patients with malignancies, diabetes, obesity, or chronic obstructive pulmonary disease (COPD) [9,10,11,12]. Moreover, in HAP, ceftobiprole warrants a good spectrum for pathogens where low to medium MDR risk is expected with the advantage in safety as compared to oxazolidinones or glycopeptides that might precipitate anemia, thrombocytopenia, and renal failure especially in frail comorbid patients [9].

In a recent experience in a multicentric Italian study, ceftobiprole was a safe and effective therapeutic choice in patients with different syndromes. It was mostly used as empiric therapy, in combination with other drugs, including carbapenems, and as a second-line therapy [13]. The aim of this single-center retrospective study is to provide a real-world experience with ceftobiprole in CAP and HAP.

## 2. Patients and Methods

An observational retrospective study has been conducted in A.O.U. Città della Scienza e della Salute, “Molinette” Hospital, Turin, Italy, including all patients with a clinical diagnosis of community-acquired pneumonia (CAP) or hospital-acquired pneumonia (HAP) according to ATS and ERS/ESICM/ESCMID/ALAT definitions [13,14,15,16] that were treated with ceftobiprole in internal medical wards (IMWs), including infectious diseases, pulmonology, and COVID-19 dedicated wards, before and during the COVID-19 pandemic. Clinical diagnosis was made with signs and symptoms and imaging; microbiological studies were recorded when available. The primary endpoints were in-hospital mortality and 30-day mortality or 3-month re-admission. Secondary endpoints evaluated were predictors of in-hospital mortality and 7-day mortality, 14-day mortality, or 30-day mortality.

### 2.1. Inclusion Criteria

All patients with a clinical diagnosis of pneumonia who received ceftobiprole for at least 48 h between 1 October 2019 and 30 June 2022, as found with the hospital’s admission and discharge databases as well as the records of the Hospital Pharmacy were included. Patients admitted to the Intensive Care Unit and surgical wards have been excluded. This study did not involve a pharmacological intervention. The treatments were always prescribed by the attending physicians according to their clinical practice.

### 2.2. Data Collection

Medical files and records have been retrospectively reviewed to confirm the diagnosis of CAP and HAP. Data collection included demographic characteristics and comorbidities (i.e., age, sex, ethnicity, days of hospitalization [dates of admission and discharge], risk factors for MRSA pneumonia or multidrug resistant (MDR) pathogens, INCREMENT-ESBL score [17], CURB-65 and PSI score [18,19], PITT score [20,21,22,23], and Charlson Comorbidity Index [24,25,26,27,28]).

Moreover, data on ceftobiprole monotherapy or combination therapy, first-line or following-lines treatment, before or following treatment with activity against ESBL or MRSA have also been collected. Primary endpoints were in-hospital mortality and 30-day mortality or 3-month re-admission. Secondary endpoints evaluated were predictors of in-hospital mortality and 7-day, 14-day, or 30-day mortality.

The definitions of the terms used In this study are as follows: Nosocomial infection: onset > 72 h after hospitalization; Sepsis/septic shock: refractory hypotension and end-organ perfusion dysfunction despite adequate fluid resuscitation; Immunedepression: congenital or acquired immunodeficiency or receipt of immunosuppressive treatment.

### 2.3. Risk Factor for MRSA

To date there are no clinical scores to predict the risk of MRSA infection or pneumonia except from local epidemiology, previous antibiotic treatments, device implants, and recent hospital stay [25]. We consider MRSA risk factors: previous MRSA colonization, presence of Gram-positive cocci in blood cultures or sputum, recent hospitalization or antibiotic treatment, necrotizing pneumonia, recent influenza, dialysis, and invasive procedures such as central-line catheter positioning or surgery.

### 2.4. INCREMENT-ESBL Score

Variables included in the INCREMENT-ESBL score were age > 50 years (OR = 2.63; 95% CI: 1.18–5.85; 3 points), infection due to *Klebsiella* spp. (OR = 2.08; 95% CI: 1.21–3.58; 2 points), source other than urinary tract (OR = 3.6; 95% CI: 2.02–6.44; 3 points), fatal underlying disease (OR = 3.91; 95% CI: 2.24–6.80; 4 points), Pitt score > 3 (OR = 3.04; 95 CI: 1.69–5.47; 3 points), severe sepsis or septic shock at presentation (OR = 4.8; 95% CI: 2.72–8.46; 4 points), and inappropriate early targeted therapy (OR = 2.47; 95% CI: 1.58–4.63; 2 points). High mortality rates (45.9%) were reported in those patients with scores ≥ 11 [17].

### 2.5. CURB-65 Score [18]

The CURB-65 can stratify the 30-day risk of mortality in patients with CAP. Included variables were: confusion; BUN > 19 mg/dL (>7 mmol/L urea); respiratory rate ≥ 30; systolic BP < 90 mmHg or diastolic BP ≤ 60 mmHg; and age ≥ 65 years old.

### 2.6. PSI Score [19]

The PSI score is able to stratify the 30-day risk of mortality in patients with CAP. Included variables were: age (years), sex, nursing home resident, neoplastic disease, liver disease history, CHF history, cerebrovascular disease history, renal disease history, altered mental status, respiratory rate ≥ 30 breaths/min, systolic blood pressure < 90 mmHg, temperature < 35 °C (95 °F) or >39.9 °C (103.8 °F), pulse ≥ 125 beats/min, pH < 7.35, BUN ≥ 30 mg/dL or ≥11 mmol/L, sodium < 130 mmol/L, glucose ≥ 250 mg/dL or ≥14 mmol/L, hematocrit < 30%, partial pressure of oxygen < 60 mmHg or <8 kPa, and pleural effusion on x-ray.

### 2.7. PITT Score [20,21,22,23]

The Pitt bacteremia score is widely used in infectious disease research as a severity of acute illness index. It ranges from 0 to 14 points, with a score ≥ 4 commonly used as an indicator of critical illness and increased risk of 30-day mortality. Included variables were: body temperature, mechanical ventilation, cardiac arrest, intravenous vasopressor, systolic blood pressure, hypotensive episode, and mental state.

### 2.8. Charlson Comorbidity Index [24,25,26,27,28]

The Charlson Comorbidity Index predicts 10-year survival in patients with multiple comorbidities. Included variables were: age, myocardial infarction; congestive heart failure, peripheral vascular disease, history of a cerebrovascular accident with minor or no residua and transient ischemic attacks, chronic cognitive deficit, COPD, connective tissue disease, history of treatment for ulcer disease or history of ulcer bleeding, liver diseases, diabetes mellitus, hemiplegia, moderate or severe chronic kidney disease, solid tumor, leukemia, lymphoma, and AIDS.

### 2.9. Statistical Analysis

Data was collected in an Excel spreadsheet and analyzed using STATASe for Mac, version 17. Continuous variables are reported as mean (standard deviation) or median (interquartile range). Categorical variables are reported as absolute number (percentage). Demographic and clinical characteristics of patients were summarized through absolute frequencies and percentages for the qualitative variables and through the percentiles (median, first quartile-third quartile) for the quantitative variables. To evaluate the primary outcome of in-hospital mortality and early re-admission from the use of ceftobiprole among CAP and HAP patients as carbapenem-, oxazolidinone-, or glycopeptide-sparing strategies, we used Cox regression and Mantel–Haenszel uni- and multi-variate models. Kaplan–Meier estimates have been used to compare overall survivals according to different use of ceftobiprole. As a secondary outcome, a logistic multivariable regression model was built to predict mortality risk selecting a priori clinically relevant predictors. Variable selection was also guided by computation of the Spearman modified correlation coefficient and knowledge of the international literature [29]. Discrimination and calibration of the model was computed. The discrimination ability was measured using the C-index. The calibration was visually assessed by evaluating the plot of the observed vs. predictive probability of death after 5000 replications using the bootstrapping procedure.

## 3. Results

Baseline characteristics are reported in Table 1. Among the 159 included patients, the majority were male (107; 67%), the median age was 70 years (IQR 60–77); there were 105 (66%) CAP and 54 (34) HAP. IMWs were the most frequent ward of admission (49; 30%), followed by hematology (29; 18%), emergency medicine (19; 12%), and cardiology (15; 9%). In thirty patients (19%) pneumonia represented a coinfection or superinfection of a concomitant COVID-19. An immune-depressing factor was present in 74 (46%) patients, as detailed in Table 1. Risk factors MRSA were a previous hospitalization within 90 days in 90 patients (56%) and a preliminary positive smear for Gram-positive cocci on sputum or bronchoalveolar lavage or blood cultures by the time of the start of ceftobiprole therapy in 34 patients (20%). The median Charlson Comorbidity Index was 5 (IQR 3–7.5) and baseline CURB-65, PSI, PITT score, and INCREMENT-ESBL have been calculated and reported in Table 1.

### 3.1. Characteristics of Treatment with Ceftobiprole

Data regarding treatment with ceftobiprole are shown in Table 2. The median duration of treatment was 10 days (IQR 7–12). First- and second-line treatments were given in 42 (26%) and 117 (74%) patients, respectively, with carbapenem-sparing regimen as the main underlying reason for the choice (i.e., low risk of ESBL etiology or de-escalation with clinical improvement after 48 h) in 71 patients (44%); other reasons were escalation for clinical severity in 14 patients, previously treated with ceftriaxone or piperacillin/tazobactam (9%), and targeted therapy after blood cultures results in 32 patients (20%).

### 3.2. Primary and Secondary Endpoints

#### 3.2.1. Risk Factors for In-Hospital Mortality and In-Hospital Mortality or 3-Month Re-Admission

Regarding primary endpoints, in-hospital mortality rate was 25% (41). At univariate analysis, risk factors for in-hospital mortality and composite outcome in-hospital mortality or 3-month re-admission are presented in Table 3. Age (HR 1.02, C.I. 1.01–1.04, *p* = 0.01), PSI (HR 1.01, C.I. 1.003–1.01, *p* = 0.003), congestive heart failure (HR 1.95, C.I. 1.15–3.2, *p* = 0.01), solid neoplasia (HR 1.23, C.I. 1.10–1.36, *p* < 0.001), and septic shock at onset (HR 5.72, C.I. 3.22–10.1, *p* < 0.0001) emerged as directly associated risk factors for in-hospital mortality. Regarding the composite outcome in-hospital mortality OR 3-month re-admission, directly associated risk factors were PSI (HR 1.40, C.I. 1.01–1.95, *p* = 0.03), congestive heart failure (HR 1.73, C.I. 1.09–2.72, *p* = 0.01), solid neoplasia (HR 1.18, C.I. 1.07–1.3, *p* < 0.001), and septic shock at onset (HR 3.49, C.I. 2.2–5.55, *p* < 0.0001). Regarding secondary endpoints, the 7-day mortality was 4% (7), the 14-day mortality was 7% (11), and the 30-day mortality was 14% (23).

In the multivariate analysis model, included variables were INCREMENT-ESBL score > 11, CAP versus HAP, COVID-19, and immunedepression. INCREMENT-ESBL score > 11 was significantly associated with incremental risk both for in-hospital mortality (HR 3.96, C.I. 2.21–7.09, *p* < 0.0001) and composite outcome (HR 3.24, C.I. 1.90–5.5, *p* < 0.0001) (Table 4).

#### 3.2.2. Overall Survival Estimates in Ceftobiprole Use

**Figure 1:**
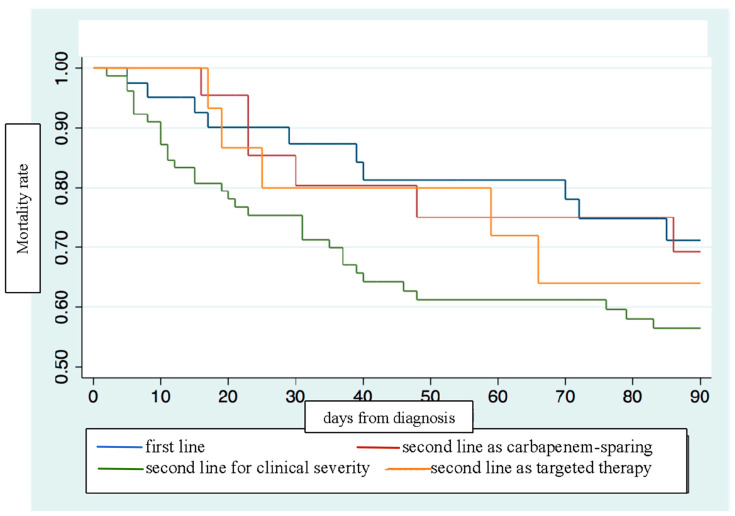
Kaplan–Meier estimates on mortality according to ceftobiprole first-line or second-line use.

**Figure 2:**
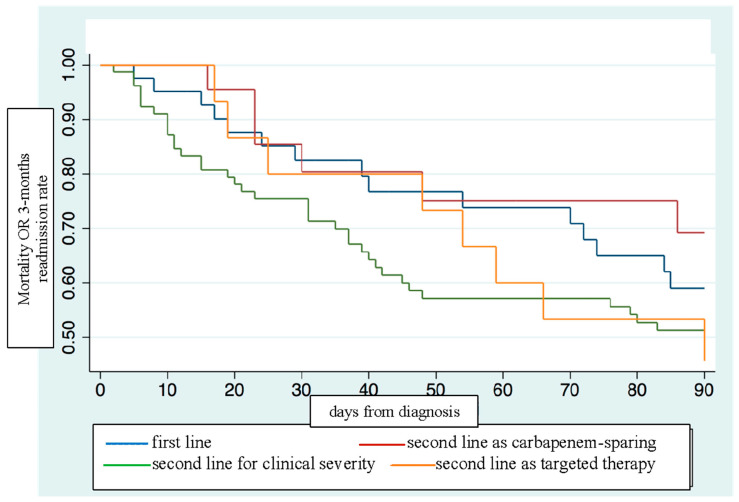
Kaplan–Meier estimates on mortality or 3-month re-admission according to ceftobiprole first-line or second-line use.

The Kaplan–Meier estimator showed how the overall survival and the composite outcome were stronger with ceftobiprole as primary therapy or as carbapenem-sparing regimen (Figure 1 and Figure 2).

However, no statistically significant differences for mortality amongst ceftobiprole use as first-line (HR 1.00, C.I. 0.46–2.17, *p* = 0.989) therapy compared to ceftobiprole use as second-line therapy [either after a carbapenem or anti-MRSA agent (HR 1.34, C.I. 0.54–3.35, *p* = 0.52) or a second line with other combination treatment (HR 0.53, C.I. 0.14–2.04, *p* = 0.364)] were reported. Moreover, no statistically significant differences were reported when a duration of treatment < 7 days was compared to a duration of treatment ≥ 7 days (HR 1.02, C.I. 0.58–1.77, *p* = 0.93).

**Figure 3:**
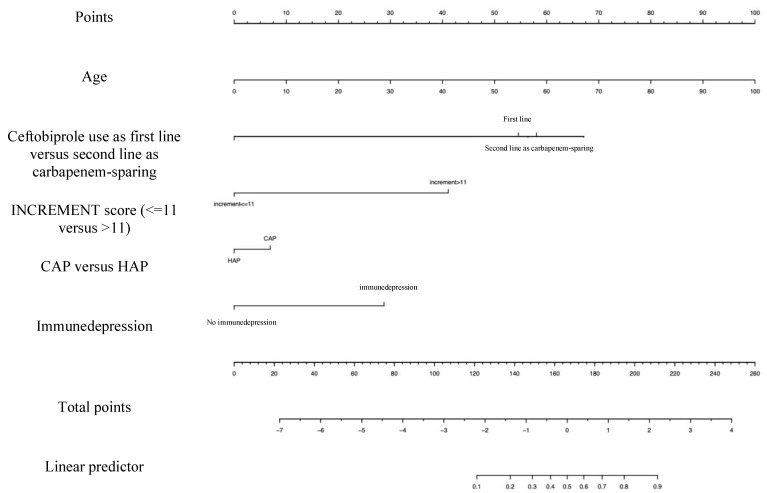
Nomogram for prognostic evaluation of risk factors for mortality.

**Figure 4:**
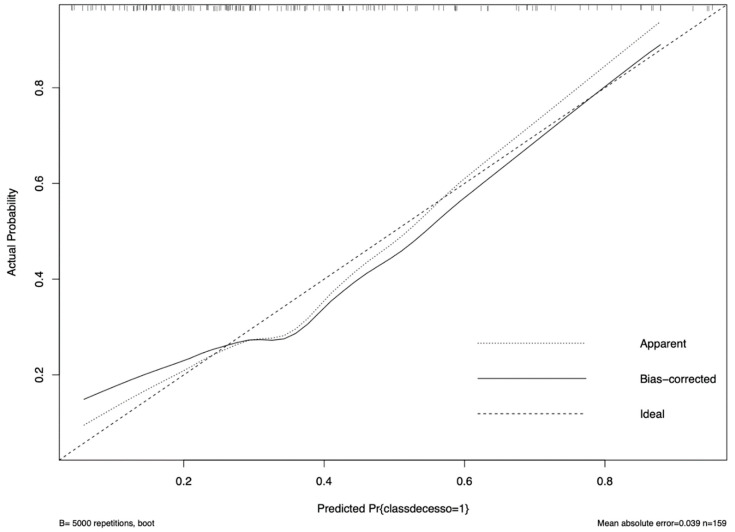
Calibration of prognostic evaluation has been conducted for internal validation.

Based on the available data, we built a prognostic model to assess which patients may benefit from sparing strategies based on nomogram risk assessment. Figure 3 is a visual tool that could help in assessing risk for mortality of each admitted patient based on some easy-to-collect variables (calibration plot is reported in Figure 4). Each variable is related to a specific score, and the sum of them (i.e., using the “points” and the “total points” lines) allows the clinician to obtain the overall probability of mortality (i.e., the bottom line, “probability”). This plot shows the differences between risk of mortality and actual events, by showing the prediction on the *x* axis and the outcome on the *y* axis. The actual data used for building the calibration are shown as small vertical lines in the upper part of the Figure 4. C-index was 72.18% [30].

## 4. Discussion

Cephalosporins have evolved greatly in the past decade through the creation of new-generation molecules with wide-spectrum activity. These new compounds theoretically permit a sparing approach in various antimicrobial classes, such as glycopeptides, lipoglycopeptides, and aminoglycosides, and they favor molecules with a good safety profile. Ceftobiprole medocaril has been approved for the treatment of adult CAP and HAP (excluding ventilator-acquired pneumonia) in 12 European countries, as well as in Canada and Switzerland.

We presented here a large real-world, single-center cohort of patients with pneumonia who were treated with ceftobiprole, which is characterized by a median age of 70 years and a median Charlson Comorbidity Index of 5, with an overall low mortality rate. Two thirds of patients had CAP. These findings, together with a majority of patients with an INCREMENT-ESBL score > 11, characterize a population with a high grade of comorbidities and with a high risk for mortality, consistent with the therapeutic choice of ceftobiprole. Few real-life studies reported ceftobiprole as a feasible option in patients with several comorbidities and polypharmacological therapies [31,32].

The majority of patients were admitted to IMWs and 20% were admitted to hematology, thus involving a proportion of immunedepressed patients. Only a small proportion of patients had concomitant COVID-19, which might have represented a predisposing factor for bacterial pulmonary co-infections or superinfections including those sustained by MDR pathogens [33,34,35]. Regarding risk factors for MRSA pneumonia, the vast majority of patients, more than 90%, had at least one recognized risk factor for MRSA as recent hospitalization, immune-depression, and recent influenza. Of note, five patients were affected by necrotizing pneumonia, of which there were three CAP cases treated with first-line ceftobiprole, and all patients survived.

Interestingly, blood cultures were performed in all cases with a 20% of positive result, which is a microbiological diagnostic rate well-described in the literature for pneumonia that usually has a low detection of pathogens [15,16,17,32]; nonetheless, the risk of performing an elevated number of blood cultures needs also to be taken into consideration.

Of note, first-line treatment with ceftobiprole was associated with better survival than escalation or targeted second-line regimens were, although there were no significant differences in the primary outcome. This is a new finding compared to other real-life studies, in which ceftobiprole in pluripathological patients was mostly used as a rescue therapy or sequential treatment [31,36].

A major feature of this study was the application of a carbapenem-sparing strategy with ceftobiprole and, indeed, the survival curves were consistent with the best results obtained with first-line treatment or carbapenem-sparing treatment. Indeed, our results represent the first evidence of ceftobiprole in this setting and it is different from the first perspective in a recent multicenter Italian study where ceftobiprole combination treatment was mainly given with carbapenems [13].

In terms of outcomes, the mortality rate was 25%, similar to another experience in which in-hospital mortality in CAP and HAP was around 30% [35]. Among baseline factors, solid tumor, congestive heart failure, and concomitant septic shock were directly associated with mortality and mortality or three-month re-admission, underlining the impact of patient comorbidities and clinical severity on outcome.

Only in a small proportion of cases clinicians associate the use of ceftobiprole with an anti-ESBL agent (8%), reflecting the confidence they have in this molecule as an alternative to carbapenems in HAP even in a setting with relatively high INCREMENT-ESBL scores; and, at the same time, considering the Italian epidemiological situation. Interestingly, we did not demonstrate a difference in mortality when ceftobiprole was used as the first line of treatment or as de-escalation from carbapenem or glycopeptide/oxazolidinone in CAP and HAP.

Moreover, even though not statistically significant, patients who received ceftobiprole as a first-line treatment or as a carbapenem-sparing strategy showed a better outcome compared to those who received ceftobiprole as a second-line regimen for any other reason. In particular, the highest excess of mortality was observed when ceftobiprole was used in as a second-line treatment for escalation for clinical severity, maybe because these patients had a poor clinical response or somehow more serious illness conditions.

Despite that the INCREMENT-ESBL score has been validated as a predictor of mortality in bloodstream infections sustained by EBSL [17], we applied, for the first time, this score in the setting of CAP-HAP and we demonstrated that an INCREMENT-ESBL score > 11 emerged as an independent predictor of both mortality and mortality or three-month re-admission. Therefore, we can speculate on the utility of the INCREMENT-ESBL, to early-identify patients who, from a stewardship perspective, may benefit either from ceftobiprole-sparring regimens, i.e., with fosfomycin, sparing either carbapenems or perhaps vancomycin or linezolid [37,38].

Lastly, we built a bedside prognostic model to assess which patients may benefit from sparing strategies based on a nomogram risk assessment. Despite the need of an external validation of this model in different cohorts, this is a visual tool that could help clinicians in assessing the risk of mortality for each admitted patient based on some easy-to-collect variables.

Several limitations of our study need to be pointed out. First, the lack of a control group and the single-center treatment experience might affect the strength of the evidence of these findings. Moreover, we did analyze CAP and HAP, which were treated with combo or monotherapy. Finally, different survival rates for treatment regimens using Kaplan–Meier estimates might have been confounded by age and comorbidity distribution, which also directly affect the outcomes.

Nonetheless, these real-life data confirm a valid role for this molecule in the treatment of CAP and HAP from a stewardship perspective and the possible use of INCREMENT-ESBL scores outside the setting of bloodstream infections. In conclusion, bearing in mind the limitations, these results could suggest a role for ceftobiprole for CAP and HAP as a stewardship tool to reduce the use of carbapenems as well as anti-MRSA agents, reducing the risk of mortality and side effects even in patients with several risk factors.

## Figures and Tables

**Table 1:** Baseline characteristics of patients (*n* = 159).

Baseline Characteristics	N (%) or Median (IQR)
Age (years)	70 (60–77)
Male gender	107 (67%)
CAP	105 (66%)
HAP	54 (34%)
Ward of admission	
Internal Medicine	49 (30%)
Hematology	29 (18%)
Emergency Medicine	19 (12%)
Cardiology	15 (9%)
Geriatrics	6 (4%)
Respiratory Medicine	5 (4%)
Other	36 (23%)
COVID-19 pneumonia	30 (19%)
Immunedepressed patients	74 (46%)
Hematological disease	33 (21%)
Immunomodulatory drugs	12 (8%)
Solid organ transplant	12 (8%)
Chemotherapy	15 (9%)
HIV	2 (1%)
Baseline risk factors for MRSA	
None	12 (7.5%)
At least one	147 (92.5%)
Colonization	9 (6%)
Previous infection	8 (5%)
Gram positive cocci on sputum/BAL	34 (21%)
Recent influenza	32 (20%)
Previous hospitalization within 90 days	90 (56%)
Necrotizing pneumonia	5 (4%)
Empyema	6 (4%)
Immunedepression	74 (46%)
Dialysis	2 (1%)
Blood cultures	
Positive	33 (21%)
Negative	126 (79%)
Not performed	0 (0%)
Broncho alveolar lavage	
Positive	20 (13%)
Negative	37 (8%)
Not performed	102 (79%)
Sputum	
Positive	5 (3%)
Negative	15 (9%)
Not performed	139 (88%)
Urinary antigen for *S. pneumoniae* and *Legionella pneumophilia*	
Positive	5 (3%)
Negative	136 (86%)
Not performed	18 (11%)
Nasal swab for MRSA	
Positive	3 (2%)
Negative	45 (28%)
Not performed	111 (70%)
Rectal swab for ESBL or CPE	
Positive	12 (8%)
Negative	70 (44%)
Not performed	77 (48%)
CURB65 in CAP	
0–1	93 (58%)
2	52 (32%)
≥3	14 (9%)
PSI in CAP	
I/II	8 (5%)
III	8 (5%)
IV	55 (34%)
V	55 (34%)
Pitt score	
<4	122 (77%)
≥4	37 (23%)
Charlson Comorbidity Index (CCI)	5 (3–7.5)
INCREMENT-ESBL Score	8 (6–11)
0–8	74 (46%)
11–14	80 (50%)
>15	5 (3%)

**Table 2:** Characteristics of treatment with ceftobiprole.

Treatment with Ceftobiprole	N (%) or Median (IQR)
Duration of treatment (days)	10 (7; 12)
First-line therapy	42 (26%)
Second-line therapy	117 (74%)
Carbapenem-sparing	71 (44%)
Escalation for clinical severity	14 (9%)
Targeted therapy	32 (20%)
Monotherapy	38 (23%)
Combination therapy with:	121 (77%)
Levofloxacin or azithromycin	94 (59%)
Anti-ESBL antibiotic, e.g., Fosfomycin	12 (8%)
Linezolid/daptomycin/vancomycin	4 (2%)
Metronidazole	11 (7%)

**Table 3:** Crude effect on mortality and mortality and 3-month re-admission.

	Crude Effect on Mortality			Crude Effect on Mortality and 3-Month Re-Admission		
	HR	*p* Value	C.I. 95%	HR	*p* Value	C.I. 95%
Age	1.02	0.01	1.01–1.04			
Male gender	1.22	0.48	0.69–2.11			
PSI/PORT	1.01	0.003	1.003–1.01	1.40	0.03	1.01–1.95
Ceftobiprole in combination therapy	0.82	0.21	0.61–1.11	0.91	0.47	0.72–1.16
Ceftobiprole as first line therapy	1.38	0.31	0.73–2.62	1.01	0.715	0.65–1.8
Positive blood cultures	1.35	0.33	0.73–2.48	1.53	0.10	0.91–2.57
C reactive protein	1.00	0.18	0.99–1.004	1.01	0.375	0.99–1.00
Procalcitonin	0.98	0.46	0.92–1.03	1.00	0.481	0.98–1.02
CAP/HAP	0.93	0.79	0.53–1.61	0.96	0.88	0.59–1.55
Immunedepression	1.007	0.91	0.87–1.15	1.00	0.99	0.88–1.13
COVID-19	0.98	0.89	0.76–1.25	1.06	0.54	0.87–1.30
Congestive heart failure	1.95	0.01	1.15–3.2	1.73	0.01	1.09–2.72
TIA/Stroke	1.69	0.19	0.76–3.76	1.71	0.13	0.85–3.46
COPD	0.95	0.88	0.50–1.81	1.03	0.11	0.59–1.77
Renal failure	1.35	0.37	0.68–2.65	1.34	0.31	0.75–2.39
Solid neoplasia	1.23	<0.001	1.10–1.36	1.18	0.001	1.07–1.3
Lymphoma	0.56	0.23	0.22–1.43	0.38	0.03	0.15–0.95
Septic shock at onset	5.72	<0.0001	3.22–10.1	3.49	<0.001	2.2–5.55

**Table 4:** Adjusted effect on mortality and mortality and 3-month re-admission.

	Adjusted Effect on Mortality			Adjusted Effect on Mortality and 3-Month Re-Admission		
	aHR	*p* Value	C.I. 95%	aHR	*p* Value	C.I. 95%
INCREMENT ESBL score > 11	3.96	<0.0001	2.21–7.09	3.24	<0.0001	1.90–5.5
CAP/HAP	0.84	0.47	0.47–1.5	0.98	0.95	0.59–1.61
COVID-19	0.93	0.71	0.71–1.21	0.98	0.83	0.78–1.22
Immunedepression	1.56	0.11	0.90–2.6	1.43	0.13	0.89–2.3

## Data Availability

Data are contained within the article.
